# Circulating metabolites associated with tumor hypoxia and early response to treatment in bevacizumab-refractory glioblastoma after combined bevacizumab and evofosfamide

**DOI:** 10.3389/fonc.2022.900082

**Published:** 2022-09-26

**Authors:** Alessia Lodi, Renu Pandey, Jennifer Chiou, Ayon Bhattacharya, Shiliang Huang, Xingxin Pan, Brandon Burgman, S. Stephen Yi, Stefano Tiziani, Andrew J. Brenner

**Affiliations:** ^1^ Department of Nutritional Sciences, The University of Texas at Austin, Austin, TX, United States; ^2^ Dell Pediatric Research Institute, Dell Medical School, The University of Texas at Austin, Austin, TX, United States; ^3^ Mays Cancer Center, The University of Texas Health Science Center at San Antonio, San Antonio, TX, United States; ^4^ Department of Oncology, Dell Medical School, Livestrong Cancer Institutes, The University of Texas at Austin, Austin, TX, United States; ^5^ Institute for Cellular and Molecular Biology (ICMB), College of Natural Sciences, The University of Texas at Austin, Austin, TX, United States; ^6^ Department of Biomedical Engineering, Cockrell School of Engineering, The University of Texas at Austin, Austin, TX, United States; ^7^ Oden Institute for Computational Engineering and Sciences (ICES), The University of Texas at Austin, Austin, TX, United States; ^8^ Department of Pediatrics, Dell Medical School, The University of Texas at Austin, Austin, TX, United States

**Keywords:** glioblastoma (GBM), bevacizumab (BEV), evofosfamide (TH-302), metabolomics (OMICS), circulating metabolites, enantiomers, D-glutamic acid (D-Glu)

## Abstract

Glioblastomas (GBM) are the most common and aggressive form of primary malignant brain tumor in the adult population, and, despite modern therapies, patients often develop recurrent disease, and the disease remains incurable with median survival below 2 years. Resistance to bevacizumab is driven by hypoxia in the tumor and evofosfamide is a hypoxia-activated prodrug, which we tested in a phase 2, dual center (University of Texas Health Science Center in San Antonio and Dana Farber Cancer Institute) clinical trial after bevacizumab failure. Tumor hypoxic volume was quantified by 18F-misonidazole PET. To identify circulating metabolic biomarkers of tumor hypoxia in patients, we used a high-resolution liquid chromatography-mass spectrometry-based approach to profile blood metabolites and their specific enantiomeric forms using untargeted approaches. Moreover, to evaluate early response to treatment, we characterized changes in circulating metabolite levels during treatment with combined bevacizumab and evofosfamide in recurrent GBM after bevacizumab failure. Gamma aminobutyric acid, and glutamic acid as well as its enantiomeric form D-glutamic acid all inversely correlated with tumor hypoxia. Intermediates of the serine synthesis pathway, which is known to be modulated by hypoxia, also correlated with tumor hypoxia (phosphoserine and serine). Moreover, following treatment, lactic acid was modulated by treatment, likely in response to a hypoxia mediated modulation of oxidative vs glycolytic metabolism. In summary, although our results require further validation in larger patients’ cohorts, we have identified candidate metabolic biomarkers that could evaluate the extent of tumor hypoxia and predict the benefit of combined bevacizumab and evofosfamide treatment in GBM following bevacizumab failure.

## Introduction

Glioblastoma (GBM) is the most common and aggressive form of primary malignant brain tumor in the adult population, with approximately 12,000 new cases diagnosed every year in the United States, and, despite modern therapies, it remains incurable (1). FDA-approved first line therapy options include temozolomide, radiation and tumor treatment fields (2). Recurrent disease uniformly develops, and salvage treatment usually includes the monoclonal antibody bevacizumab (Bev). Bev depletes vascular endothelial growth factor, a hypoxia induced factor (HIF)-driven gene which promotes tumoral angiogenesis and neovascularization. Yet, while bevacizumab typically results in radiographic responses, it has not significantly improved overall survival. Resistance to Bev has been shown to be driven by tumor hypoxia (3). Evofosfamide (Evo or TH302) is a hypoxia-activated nitroimidazole prodrug, which, when exposed to hypoxia, is reduced by intracellular reductases and releases the alkylating agent bromo-isophosphoramide mustard (Br-IPM). Br-IPM, a cytotoxin, can then crosslink cellular DNA (4–8). Our phase 2, dual center (University of Texas Health Science Center in San Antonio and Dana Farber Cancer Institute) clinical trial investigated the outcome of combined Bev/Evo in 33 Bev refractory GBM patients (5). The combined Bev/Evo treatment resulted in a statistically significant improvement over previous therapies, with a 31% progression free survival at 4 months (PFS-4). Moreover, hypoxia volume (HV) was calculated and both progression free survival and overall survival were negatively associated with hypoxia volume values (9).

Modulation of cell metabolism is a well-established feature that cancer cells use to thrive and is reflected intracellularly, in the tumor microenvironment, and in the levels of circulating metabolites. Metabolic profiling studies are typically performed using platforms based on mass spectrometry or magnetic resonance spectroscopy to characterize an ever-increasing number of metabolites in biosamples including cells, tissues and biofluids (10–18). Circulating metabolites are increasingly being investigated as potential biomarkers for disease detection (11, 19) and progression (20), survival prediction (21) and response to treatment (22). In addition, metabolite stereospecificity recognition is key in the identification of specific metabolic biomarkers in health and disease (23–33). The predominance of a specific amino or hydroxy acid stereoisomer form has been recognized in cancer as well as in other metabolic diseases. For instance, the relative levels of D- and L-2-hydroxyglutaric acid in cancer have been shown to depend on the mutation status of isocitrate dehydrogenase 1 and 2, and cellular hypoxia (34–38). To improve the detection of stereoisomers in biosamples, we have recently developed an untargeted, LC-MS-based method for the simultaneous detection of different classes of metabolites, including hydroxy and amino acids, in a single analytical run (39).

Here, we investigated the profiles of the circulating metabolites in patients with recurrent glioblastoma following Bev failure to evaluate candidate metabolic biomarkers associated with hypoxia in the tumor. In addition, on a very small number of patients, we also evaluated the association between patient specific metabolic changes observed early and at later stages of treatment to identify potential biomarkers of response to treatment.

## Materials and methods

### Patients and clinical trial design

All information about the patient characteristics, the criteria for admission in the clinical trial and the clinical trial design are included in (5). Briefly, 33 patients, ages 19 to 76 years (median age: 47 years) with progressive or recurrent glioblastoma following Bev were considered in the study. MGMT status was methylated in 26%, unmethylated in 32%, and unknown in 42%. IDH mutations were identified in 29%, not seen in 46%, and unknown in 25%. The clinical trial (phase 2, open label, single arm) evaluated Bev (10 mg/kg) in combination with Evo (670 mg/m2), following Bev failure. All patients had previously received radiation therapy and temozolomide chemotherapy, as well as Bev. Overall survival was defined as the interval from the start of Bev/Evo treatment until death. For all patients, serum was collected shortly prior to receiving the first dose of the combined Bev/Evo and with each cycle of therapy. The protocol was approved by the institutional review board at the University of Texas Health Science Center at San Antonio (UT) and at the Dana Farber Cancer Institute (DF). All patients provided written informed consent. All methods were carried out in accordance with Good Clinical Practice and in accordance with local guidelines and regulations. This trial was registered with www.clinicaltrials.gov (NCT02342379) on 19 Jan 2015.

### Calculation of tumor hypoxia by FMISO-PET

In all cases, patients were injected intravenously with 3.7 MBq/kg of 18F-FMISO. A 20-minute static 18F-FMISO PET emission image was acquired at about 120 minutes after injection of 18F-FMISO. PET scans were performed on two devices, both of which were calibrated. On a CTI EXACT HR+ scanner and a Siemens Biograph40 mCT scanner as previously described (8). The tumor ROIs on the 18F-FMISO PET images included all regions where there was FMISO uptake, and two 2 cm diameter ROIs on both sides of the cerebellar cortex were used as the image derived blood surrogate to determine the surrogate of tissue to blood ratio (TB ratio). HV was determined by the number of pixels with TB ratio above 1.2.

### Sample preparation for metabolic analysis

Serum samples were initially collected between June 2015 and August 2017 and stored in liquid nitrogen until analysis. Thirty serum samples were analyzed for the cycle 1 timepoint (prior to first dose of treatment). 10 serum samples were analyzed for the cycle 2 and end of treatment (EOT or cycle 5) timepoints. Additional sample details are included in **Supplementary Data Table**.

Plasma samples were thawed on ice. For each plasma sample, a volume of 200 µL was transferred to washed Nanosep 3K Omega centrifugal filters (Pall Corporation, Port Washington, NY, USA) and centrifuged for 24 hours at 8,000 rpm and at 4°C (16, 17, 40). The plasma filtrate (polar fraction) was recovered and split into two parts for the untargeted analyses of the polar metabolites and metabolite enantiomeric forms (chiral metabolomics).

### Reagents

LC-MS grade water, methanol, acetonitrile, formic acid, ammonium acetate, ammonium formate, 2,6-di-tert-butyl-4-methylphenol (BHT) were used for the analysis (Thermo Fisher Scientific, Waltham, MA, USA). Commercial calibration solutions for the mass spectrometer were also purchased from Thermo Fisher Scientific (Waltham, MA, USA).

### Untargeted polar metabolomics

For the untargeted polar metabolite profiling, the plasma filtrate was diluted in water (1:500 ratio) and transferred to LC-MS vials for analysis as previously described (16, 18). Pooled quality control samples were injected every 6-th sample. The metabolic profiling analysis was conducted on the Accela 1253 UPLC system with a quaternary pump in tandem with a Hybrid Quadrupole Orbitrap mass spectrometer (Q Exactive, Thermo Scientific, Bremen, Germany) equipped with electrospray ionization (ESI) operating in negative/positive ion switching mode (Thermo Fisher Scientific, San Jose, CA, USA). The chromatographic separation of metabolites was achieved *via* a Synergi 4 µm Hydro-RP 80 Å, 150 × 2 mm HPLC column (Phenomenex, Torrance, CA, USA). Mobile phases (A) HPLC water and (B) methanol were run at 99/1 for 2 minutes, then increased linearly from 70/30 to 20/80 in 8 minutes, with a wash of 2/98 for 5 minutes and a column equilibration time of 15 minutes. The total run time was 30 minutes with 5 µL injection volume and 250 µL/min flow rate, as previously described (18). Detection was completed in full MS mode with the following settings: spray voltage, 3.5 kV; capillary temperature, 320°C; sheath gas, 45 (arbitrary units); auxiliary gas, 10 (arbitrary units); m/z range, 70‐1000 (HILIC), 50 to 750 (RP); data acquisition, centroid mode; microscans, 10; AGC target, 1e6; maximum injection time, 200 ms; mass

### Chiral metabolomics analysis

Chiral metabolomics was conducted on the Vanquish Flex UHPLC (Fisher Scientific, San Jose, CA, USA) equipped with an ACQUITY BEH C18 150 × 2.1 mm (1.7 μm, 130 Å) column (Waters, Milford, MA) in tandem with the Q Exactive Hybrid Quadrupole Orbitrap mass spectrometer (Thermo Scientific, Bremen, Germany) equipped with electrospray ionization (ESI) operating in negative/positive ion switching mode. Mobile phases (A) 0.06% formic acid and 10mM ammonium formate, (B) 0.1% formic acid in acetonitrile, (C) 0.1% formic acid in methanol were run at a ratio of 98/1/1 to 90/5/5 for 14 minutes, 90/5/5 to 95/5/0 from 14-14.5, 95/5/0 to 92/8/0 from 14.5-31 minutes, 92/8/0 to 67/33/0 from 31-62 minutes, 67/33/0 to 98/1/1 from 62-63 minutes with an equilibration time of 12 minutes. Total run time was 75 minutes. Quality control samples were made prior to undergoing derivatization by (+)-diacetyl-L-tartaric anhydride and (-)-diacetyl-D-tartaric anhydride as previously described (39). Detection was completed in full MS mode in positive ionization mode with the following settings: spray voltage, 4.0 kV; capillary temperature, 320°C; sheath gas, 45 (arbitrary units); auxiliary gas, 10 (arbitrary units); S-lens RF-level, 50; micro scans, 1; AGC target, 1e6; maximum injection time, 200 ms; mass resolution, 70,000/35,000 fwhm; m/z range, 70–1000. Pooled quality control samples were injected every 6-th sample.

### Data processing and statistical analysis

SIEVE 2.2.0 SP2 software (Thermo Scientific, San Jose, CA, USA) was used to conduct peak picking and spectral alignment on raw data. Peak identities were assigned by matching the mass-to-charge ratio and retention time values to an in-house library of compounds. Peaks with a coefficient of variation (CV) greater than 25% in the pooled quality control repeat injections were excluded from the analysis. The Pearson and Spearman correlation coefficients and their significance were calculated to study the association between metabolite levels and survival data using Matlab. A False Discovery Rate correction was applied to p-values obtained from the correlation analysis and q-values <0.05 were considered significant. We conducted 100,000 sample permutation statistical tests to sample all patients.

## Results

### Peripheral blood metabolites as markers of tumor hypoxia

We investigated whether specific metabolites in the peripheral circulation were associated with the extent of tumor hypoxia evaluated in GBM patients *via* imaging techniques prior to the first dose of combined Bev/Evo. Blood plasma samples were profiled using untargeted, high resolution mass spectrometry (aimed at detecting a large number of metabolites) combined with a newly developed approach to differentiate the specific enantiomeric forms of amino and hydroxy acids, also in an untargeted fashion. 130 unique metabolites and 60 enantiomeric forms (30 enantiomeric pairs) were identified based on matching mass-to-charge ratio and retention time values to an in-house library of compounds.

**Figure 1:**
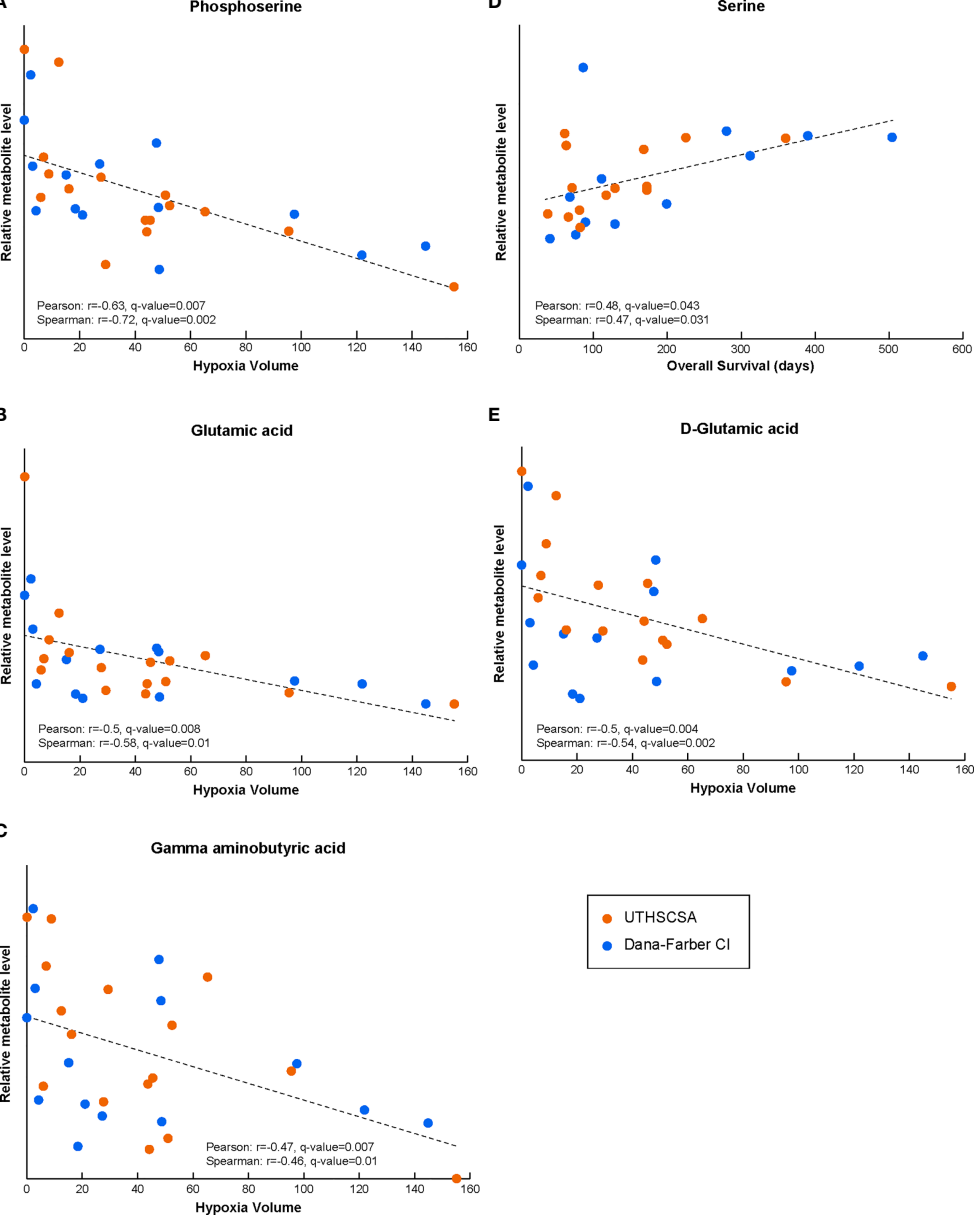
The levels of circulating metabolites in Bevacizumab-refractory GBM patients prior to treatment with Bev/Evo correlate with tumor hypoxia and OS. Phosphoserine, and gamma aminobutyric, glutamic and D-glutamic acids circulating levels result in significant correlation with HV **(A–C, E)**. Serine levels significantly correlated with OS **(D)**. In all panels, the Pearson correlation linear regression is shown with the black dashed line. Datapoints for each patient are shown in orange for patients enrolled at the University of Texas Health Science Center in San Antonio (UTHSCSA; 16 and 14 patient samples for HV and OS correlations, respectively) and in blue for patients enrolled at Dana-Farber Cancer Institute (CI; 14 and 12 patient samples for HV and OS correlations, respectively). Evaluation of significance included corrections for repeated measurements and sample permutation (100,000 permutations) statistical tests (included in **Supplementary Figure 1**).

We correlated the blood metabolite levels and patients’ HV to investigate any association between specific circulating metabolites and the extent of tumor hypoxia. The results below include metabolites that demonstrate significant Pearson and Spearman correlations with patients’ hypoxia in the tumor (hypoxia volume, HV) or survival. Blood samples from 30 patients (16 and 14 collected at UT and DF, respectively) with matched HV levels were available for this analysis. Our correlation of blood resulted in several circulating metabolites with significant associations. Serum levels of phosphoserine (Pearson r=-0.63, q-value=0.007; Spearman r=-0.72, q-value=0.002), glutamic acid (Pearson r=-0.50, q-value=0.008; Spearman r=-0.58, q-value=0.01) and gamma-aminobutyric acid (Pearson r=-0.47, q-value=0.007; Spearman r=-0.46, q-value=0.01) all resulted in significant correlations with HV (**Figure 1A–C** and **Supplementary Figure 1A–C**). Interestingly, the blood level of serine, a metabolite very closely related to phosphoserine, also resulted in a significant correlation with patient’s survival data (**Figure 1D** and **Supplementary Figure 1D**; Pearson r=0.48, q-value=0.043; Spearman r=0.47, q-value=0.031). Phosphoserine, glutamic acid and gamma-aminobutyric acid, while significantly correlated to HV, were not correlated to OS (data in **Supplementary Data Table 1**). Other detected metabolites in the glycolysis and serine pathways did not correlate to either HV or OS (data in **Supplementary Data Table 2**). Blood samples from 26 patients (14 and 12 collected at UT and DF, respectively) with matched OS data were available for this analysis (**Supplementary Data Tables**; for some patients with available HV levels, OS data were unknown),.

Moreover, the analysis of the correlation between the HV data and the levels of the specific enantiomeric forms of amino and hydroxy acids resulted in a significant correlation coefficient for D-glutamic acid (Pearson r=-0.50, q-value=0.004; Spearman r=-0.54, q-value=0.002; **Figure 1E** and **Supplementary Figure 1E**), but not L-glutamic acid (not shown; both forms trended similarly vs HV and did not correlate with OS).

### Peripheral blood metabolites as markers of response

Patients’ blood samples were collected immediately prior to treatment and at several times points during treatment. To identify blood metabolites that might offer insight into the patient’s response to treatment, we investigated whether the extent of the metabolite level changes during treatment in matched patient samples (compared to prior to the start of treatment) correlated with HV and/or survival. More specifically, for each patient, the ratio of the metabolite levels at a given time during treatment (either cycle 2 or cycle 5 of treatment) and the metabolite level prior to treatment was calculated, and correlations with HV or survival were evaluated. These analyses were limited by the small number of samples available, specifically 10 samples for both the cycle 2 timepoint (7 from DF and 3 from UT), and end of treatment (cycle 5; 2 from DF and 8 from UT).

**Figure 2:**
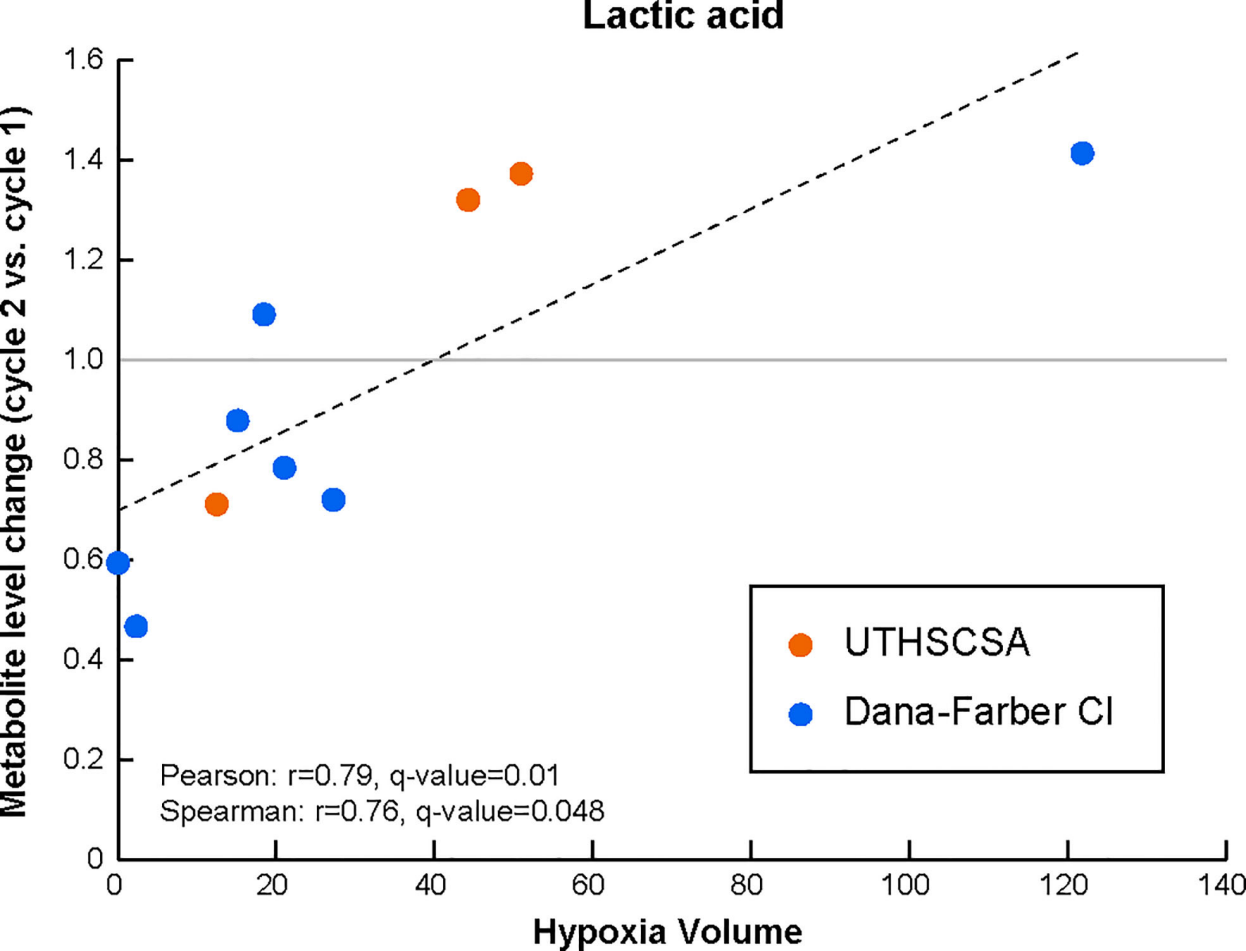
Lactic acid levels in the peripheral circulation are modulated early during treatment with Bev/Evo to an extent that is associated with tumor hypoxia levels prior to treatment. Changes in the levels of the blood metabolite after treatment (i.e. levels at cycle 2 normalized to the matched levels prior to treatment) significantly correlate with HV. The Pearson correlation linear regression is shown with the black dashed line. Datapoints for each patient are shown in orange for patients enrolled at the University of Texas Health Science Center in San Antonio (UTHSCSA; 3 patient samples) and in blue for patients enrolled at Dana-Farber Cancer Institute (CI; 7 patient samples). Evaluation of significance included corrections for repeated measurements and sample permutation (100,000 permutations) statistical tests (included in **Supplementary Figure 1**).

Interestingly, and although the number of patient samples available for this analysis was very limited, changes lactic acids serum levels after cycle 2 of treatment (as compared to the levels before treatment) significantly correlated with HV (Pearson r=0.79, q-value=0.01; Spearman r=0.76, q-value=0.048; **Figure 2** and **Supplementary Figure 1F**).

No significant correlations were identified between HV before treatment and changes in serum metabolite levels at the end of treatment (cycle 5 compared to before treatment).

### Peripheral blood metabolites do not reflect MGMT or IDH status

We investigated the presence of associations between circulating metabolite levels and MGMT or IDH status in our GBM patient cohort. MGMT status was methylated in 26%, unmethylated in 32%, and unknown in 42% of the patients. IDH mutations were identified in 29%, not seen in 46%, and unknown in 25%. Metabolite levels in the peripheral circulation were found not to reflect either MGMT or IDH status.

## Discussion

The combination of Evo and Bev was evaluated in recurrent glioblastoma patients following Bev failure in a dual center, phase 2 trial (5). As previously reported, the Bev/Evo combination resulted in improved outcomes in patient’s PFS. A number of brain imaging readings (such as hypoxic volume, perfusion and anatomic radiographic features) were evaluated to determine if any of these could provide a non-invasive mean to predict the benefits of the combined Bev/Evo treatment (9). Based on those measurements, the hypoxic volume in the tumor was determined to be negatively associated with PFS and OS (9).

Even though the imaging parameters provide a direct reflection of the localized status near the tumor area, the multivariate nature of the metabolic profiling in the peripheral circulation has the potential to provide complementary information to identify patients that could benefit from this regimen following Bev failure.

The metabolic profiling was performed using untargeted, high-resolution LC-MS-based metabolomics approaches to profile total pool metabolite levels as well as the relative levels of the enantiomeric forms of amino and hydroxy acid compounds. These include, for example, the D- form of amino acids (e.g., D-serine). While the D- amino acids have been deemed “unnatural” in the past and are still generally not receiving much attention in human studies, we and others have shown that these metabolites are present not only in human biofluids (possibly deriving from the microbiome) but also in human cell lines (in some cases at levels comparable to the “natural” L- enantiomeric form) and are important in the search of metabolic biomarkers to evaluate aspects including disease development and progression, and response to treatment (23–25, 27–32, 37–39).

Here, we investigated (i) whether the blood level of metabolites in the peripheral circulation could reflect the severity of hypoxia in the tumor and offer an alternative or additional way to assess hypoxia levels in the tumor and (ii) whether the changes in circulating metabolites during treatment could provide early response information to evaluate treatment efficacy. Given the limited number of patients involved in this study, the candidate metabolic biomarkers we report here will need to be further validated.

Among several other circulating metabolites, phosphoserine serum levels prior to treatment significantly correlated with HV. In addition, serine levels before treatment were positively correlated with OS. In the serine synthesis pathway, phosphoserine is formed from 3-phosphoglycerate *via* phosphoglycerate dehydrogenase (PHGDH) and phosphoserine aminotransferase 1 (PSAT1). Phosphoserine phosphatase (PSPH) then converts phosphoserine to serine. Hypoxia has been shown to induce expression of PHGDH, PSAT1 and PSPH in breast cancer cells (41) and PHGDH has been shown to be overexpressed in various cancer types, including colorectal, non-small cell lung, cervical and breast cancers (42–44). Taken together, the blood level of phosphoserine and serine suggest the potential usefulness of these circulating metabolites in accessing the extent of tumor hypoxia and the identifying patients that could benefit from combined Bev/Evo following Bev failure.

In addition to phosphoserine, also glutamic and gamma aminobutyric acid blood levels prior to treatment were both negatively correlated with HV. In addition, the enantiomeric analysis of the relative amounts of stereoisomer compounds, revealed that D-glutamic acid, but not L-glutamic acid, significantly negatively correlated with HV at diagnosis. Glutamic acid and gamma aminobutyric acid are both key metabolites/neurotransmitters in the normal brain (45, 46). Active neurons have been shown to impact glioma growth and progression (46, 47), therefore, while their function and modulation in glioblastomas and hypoxia has not been clarified, one could speculate that these neurotransmitter metabolites might also have key roles in glioblastoma progression and growth. Moreover, glioma cells and many other cancer cell types, are highly dependent on glutamine (from which glutamic acid is derived) for their heightened biosynthetic and energetic needs (48, 49). Glutamic acid and gamma aminobutyric acid are closely related to D-glutamic acid which points to the potential of the enantiomeric form to further improve the relevance of metabolic signatures of disease.

Interestingly, the magnitude of the changes in the blood levels of lactic acid early on during treatment (compared to prior to treatment) were positively associated with the pre-treatment extent of hypoxia in the tumor. Hypoxia was shown to induce transcription of glycolytic enzymes mediated by HIF-1, including activation of pyruvate dehydrogenase kinase 1 (PDK1) and lactate dehydrogenase A (LDHA) leading to a switch in cell metabolism to glycolysis (50). The observed changes might therefore reflect changes in tumor hypoxia following treatment and, if confirmed in larger studies could serve as a means of non-invasively following treatment response and efficacy.

Given the lack of effective circulating tumor markers for glioblastoma and the known issues in radiographic assessment of response including pseudo-response and pseudo-progression, the potential of serum metabolites as ancillary markers of response is clinically significant. Additional studies are needed that include a larger number of patients to confirm the finding that the levels of these circulating metabolites represent a reflection of tissue hypoxia, possible hypoxia biomarkers and predictors of treatment efficacy.

In summary, as a general finding our study highlights the importance to consider metabolic profiles in human biosamples that include the specific enantiomeric forms of various metabolites to further improve our ability to discover novel multivariate metabolic signatures of disease and treatment response. Specifically, as it relates to the Bev/Evo treatment being considered here, the plasma levels of circulating intermediates related to serine synthesis pathway and glycolysis prior to treatment and their changes during treatment might provide important indicators associated with tissue hypoxia and valuable predictors of the efficacy of the combined Bev/Evo treatment.

## Data availability statement

The original contributions presented in the study are included in the article/**Supplementary Material**. Further inquiries can be directed to the corresponding authors.

## Ethics statement

The studies involving human participants were reviewed and approved by institutional review board at the University of Texas Health Science Center at San Antonio and at the Dana Farber Cancer Institute. The patients/participants provided their written informed consent to participate in this study.

## Author contributions

All authors listed have made a substantial, direct, and intellectual contribution to the work and approved it for publication.

## Funding

This study was supported by an FDA Orphan Products Research Project Grant (TH-302, R01FD004400).

## Acknowledgments

We are grateful to the patients who participated in this study. SY was supported by the National Institutes of Health grant R35GM133658.

## Conflict of interest

The authors declare that the research was conducted in the absence of any commercial or financial relationships that could be construed as a potential conflict of interest.

## Publisher’s note

All claims expressed in this article are solely those of the authors and do not necessarily represent those of their affiliated organizations, or those of the publisher, the editors and the reviewers. Any product that may be evaluated in this article, or claim that may be made by its manufacturer, is not guaranteed or endorsed by the publisher.
