# Supplementary material for: Circulating metabolites associated with tumor hypoxia and early response to treatment in bevacizumab-refractory glioblastoma after combined bevacizumab and evofosfamide
Source: Front Oncol. 2022 Sep 26;12:900082. doi: 10.3389/fonc.2022.900082 (PMC9549210; doi:10.3389/fonc.2022.900082)
Supplement: Supplementary file 1 [file DataSheet_1.docx]

**Supplementary Data.**

**Table 1.** Hypoxic volume (HV), overall survival (OS) and relative circulating metabolite levels of selected metabolites immediately prior to the beginning of treatment (cycle 1), at cycle 2 and at the end of treatment (EOT) for patients enrolled in the clinical trial. DF### and UT### indicate patients enrolled at Dana Farber Cancer Institute and University of Texas Health Science Center in San Antonio, respectively. Data for glutamic acid also includes the percentages of the L- and D-enantiomeric forms. (N.A.: survival data not available; N.D.: not detected)

|  | **HV** | **OS** | **Gamma-Aminobutyric acid** | | | **Phosphoserine** | | | **Lactic acid** | | |
| --- | --- | --- | --- | --- | --- | --- | --- | --- | --- | --- | --- |
|  |  |  | **Cycle 1** | **Cycle 2** | **EOT** | **Cycle 1** | **Cycle 2** | **EOT** | **Cycle 1** | **Cycle 2** | **EOT** |
| **DF001** | 18.43 | 280 | 18510 | 73045 |  | 746251 | 630323 |  | 2437862 | 2657113 |  |
| **DF002** | 121.85 | 129 | 39393 | 147668 |  | 480923 | 950338 |  | 1617420 | 2284357 |  |
| **DF003** | 27.24 | 68 | 35952 | 40235 |  | 1003411 | 737654 |  | 2960301 | 2130401 |  |
| **DF004** | 144.82 | N.A. | 31897 |  |  | 531891 |  |  | 3053841 |  |  |
| **DF005** | 97.51 | 41 | 65986 |  | 74526 | 715005 |  | 536026 | 4538459 |  | 4596614 |
| **DF006** | 0.03 | N.A. | 92390 | 354508 |  | 1254709 | 2305349 |  | 3502927 | 2075414 |  |
| **DF007** | 15.12 | 504 | 66545 | 26125 |  | 940336 | 373164 |  | 2599224 | 2281296 |  |
| **DF008** | 21.02 | 199 | 42828 | 57050 |  | 710330 | 370968 |  | 3440342 | 2695267 |  |
| **DF009** | 47.69 | 390 | 125797 |  | 35611 | 1123782 |  | 332155 | 1908306 |  | 2957884 |
| **DF011** | 48.71 | 76 | 32696 |  |  | 398305 |  |  | 2761907 |  |  |
| **DF013** | 48.43 | 111 | 102083 |  |  | 753714 |  |  | 4620577 |  |  |
| **DF014** | 4.27 | 89 | 45189 |  |  | 735184 |  |  | 3809049 |  |  |
| **DF016** | 2.32 | 312 | 154998 | 48759 |  | 1515038 | 726551 |  | 5069048 | 2361735 |  |
| **DF019** | 3.02 | 86 | 109225 |  |  | 991318 |  |  | 2134369 |  |  |
| **UT001** | 155.11 | 63 | N.D. |  |  | 298397 |  |  | 6434201 |  |  |
| **UT002** | 95.46 | 168 | 61737 |  | 34297 | 617414 |  | 463218 | 2494145 |  | 3840520 |
| **UT003** | 52.44 | 61 | 88024 |  | 117411 | 764028 |  | 934732 | 2981324 |  | 2312198 |
| **UT005** | 7.01 | 129 | 122025 |  |  | 1042706 |  |  | 2884091 |  |  |
| **UT006** | 29.38 | 225 | 108570 |  | 40720 | 426621 |  | 257930 | 2419919 |  | 1832071 |
| **UT007** | 44.24 | 71 | 16827 | N.D. |  | 613741 | 319173 |  | 3156597 | 4164974 |  |
| **UT008** | 8.85 | 117 | 149114 |  |  | 946215 |  |  | 3766768 |  |  |
| **UT013** | 65.24 | 172 | 115781 |  | 334721 | 729008 |  | 1807933 | 3011582 |  | 5862722 |
| **UT015** | 45.49 | 172 | 57844 |  |  | 680104 |  |  | 5257288 |  |  |
| **UT016** | 0.08 | N.A. | 150090 |  | 161411 | 1661645 |  | 2823797 | 4632422 |  | 3452159 |
| **UT017** | 16.10 | 38 | 82751 |  | 31144 | 861184 |  | 1336152 | 5597753 |  | 3354417 |
| **UT018** | 5.99 | 360 | 53001 |  |  | 812211 |  |  | 1850333 |  |  |
| **UT019** | 50.94 | 81 | 23108 | 95445 |  | 824001 | 1285258 |  | 3471282 | 4760874 |  |
| **UT020** | 27.67 | 66 | 44116 |  | N.D. | 927212 |  | 474892 | 4071355 |  | 5646637 |
| **UT021** | 43.71 | 82 | 54359 |  | 184256 | 680275 |  | 1993988 | 4485862 |  | 4898442 |
| **UT022** | 12.47 | N.A. | 96283 | 160806 |  | 1587793 | 1511884 |  | 2522357 | 1791859 |  |

**Table 1.** continued

|  | **HV** | **OS** | **Serine** | | | **Glutamic acid** | | | | |
| --- | --- | --- | --- | --- | --- | --- | --- | --- | --- | --- |
|  |  |  | **Cycle 1** | **Cycle 2** | **EOT** | **Cycle 1** | **Cycle 2** | **EOT** | **%L-form** | **%D-form** |
| **DF001** | 18.43 | 280 | 3973662 | 2922209 |  | 26064011 | 32972024 |  | 51.5 | 48.5 |
| **DF002** | 121.85 | 129 | 2197577 | 2862642 |  | 29938858 | 49140664 |  | 39.9 | 60.1 |
| **DF003** | 27.24 | 68 | 2712463 | 2615293 |  | 43135034 | 29089045 |  | 45.8 | 54.2 |
| **DF004** | 144.82 | N.A. | 2325538 |  |  | 22252989 |  |  | 10.4 | 89.6 |
| **DF005** | 97.51 | 41 | 1916809 |  | 1427786 | 31058526 |  | 27956993 | 45.0 | 55.0 |
| **DF006** | 0.03 | N.A. | 3223012 | 3611544 |  | 63797648 | 79595492 |  | 41.5 | 58.5 |
| **DF007** | 15.12 | 504 | 3855594 | 2230634 |  | 39252592 | 18336908 |  | 38.4 | 61.6 |
| **DF008** | 21.02 | 199 | 2581171 | 2325002 |  | 24406197 | 25393320 |  | 51.7 | 48.3 |
| **DF009** | 47.69 | 390 | 3885472 |  | 2692456 | 43533928 |  | 21572526 | 25.9 | 74.1 |
| **DF011** | 48.71 | 76 | 1993553 |  |  | 24883152 |  |  | 39.5 | 60.5 |
| **DF013** | 48.43 | 111 | 3061707 |  |  | 42244622 |  |  | 9.4 | 90.6 |
| **DF014** | 4.27 | 89 | 2232942 |  |  | 29902683 |  |  | 39.2 | 60.8 |
| **DF016** | 2.32 | 312 | 3503070 | 2323154 |  | 70088509 | 53033078 |  | 25.2 | 74.8 |
| **DF019** | 3.02 | 86 | 5191111 |  |  | 50894688 |  |  | 48.4 | 51.6 |
| **UT001** | 155.11 | 63 | 3699972 |  |  | 22214065 |  |  | 36.6 | 63.4 |
| **UT002** | 95.46 | 168 | 3625219 |  | 4488622 | 26563174 |  | 27890886 | 43.5 | 56.5 |
| **UT003** | 52.44 | 61 | 3927917 |  | 2563578 | 38739032 |  | 43332015 | 42.8 | 57.2 |
| **UT005** | 7.01 | 129 | 2881760 |  |  | 39547520 |  |  | 10.7 | 89.3 |
| **UT006** | 29.38 | 225 | 3850264 |  | 2189425 | 27428794 |  | 14929908 | 9.9 | 90.1 |
| **UT007** | 44.24 | 71 | 2889359 | 2466748 |  | 29959254 | 13572452 |  | 11.2 | 88.8 |
| **UT008** | 8.85 | 117 | 2749083 |  |  | 46808324 |  |  | 11.6 | 88.4 |
| **UT013** | 65.24 | 172 | 2845994 |  | 3445005 | 40759914 |  | 89233998 | 33.6 | 66.4 |
| **UT015** | 45.49 | 172 | 2916233 |  |  | 38148957 |  |  | 11.4 | 88.6 |
| **UT016** | 0.08 | N.A. | 2338149 |  | 3603095 | 109202784 |  | 100706615 | 49.4 | 50.6 |
| **UT017** | 16.10 | 38 | 2392032 |  | 1344622 | 41884875 |  | 49817171 | 40.5 | 59.5 |
| **UT018** | 5.99 | 360 | 3839497 |  |  | 35216963 |  |  | 11.7 | 88.3 |
| **UT019** | 50.94 | 81 | 2462706 | 2388040 |  | 30769729 | 43530754 |  | 25.4 | 74.6 |
| **UT020** | 27.67 | 66 | 2332080 |  | 2041408 | 36127805 |  | 19953726 | 7.4 | 92.6 |
| **UT021** | 43.71 | 82 | 2132555 |  | 2470000 | 26091832 |  | 67535628 | 26.6 | 73.4 |
| **UT022** | 12.47 | N.A. | 3034333 | 2738109 |  | 57028196 | 49930990 |  | 11.3 | 88.7 |

**Table 2.** Hypoxic volume (HV), overall survival (OS) and relative circulating metabolite levels of selected metabolites immediately prior to the beginning of treatment (cycle 1), at cycle 2 and at the end of treatment (EOT) for patients enrolled in the clinical trial. DF### and UT### indicate patients enrolled at Dana Farber Cancer Institute and University of Texas Health Science Center in San Antonio, respectively.

|  | **HV** | **OS** | **Pyruvic acid** | | | **Glucose** | | | **Glycine** | | |
| --- | --- | --- | --- | --- | --- | --- | --- | --- | --- | --- | --- |
|  |  |  | **Cycle 1** | **Cycle 2** | **EOT** | **Cycle 1** | **Cycle 2** | **EOT** | **Cycle 1** | **Cycle 2** | **EOT** |
| **DF001** | 18.43 | 280 | 83359756 | 53584656 |  | 42614794 | 53966671 |  | 4493108 | 4643011 |  |
| **DF002** | 121.85 | 129 | 53220931 | 73334359 |  | 44121450 | 48108857 |  | 2164243 | 1937400 |  |
| **DF003** | 27.24 | 68 | 75811471 | 58608908 |  | 81578557 | 36128511 |  | 2541933 | 2554808 |  |
| **DF004** | 144.82 | N.A. | 71031042 |  |  | 31814827 |  |  | 3204092 |  |  |
| **DF005** | 97.51 | 41 | 126775871 |  | 133077479 | 50749342 |  | 43048872 | 2916540 |  | 3551835 |
| **DF006** | 0.03 | N.A. | 125054176 | 62890981 |  | 77691228 | 92626494 |  | 2934533 | 3598070 |  |
| **DF007** | 15.12 | 504 | 62382374 | 38911524 |  | 50220399 | 41035736 |  | 2000128 | 2784385 |  |
| **DF008** | 21.02 | 199 | 54206062 | 56390442 |  | 70630232 | 48607388 |  | 2370006 | 1965000 |  |
| **DF009** | 47.69 | 390 | 48483545 |  | 72249376 | 48274479 |  | 74282113 | 5447400 |  | 4966483 |
| **DF011** | 48.71 | 76 | 62279207 |  |  | 54233298 |  |  | 2393246 |  |  |
| **DF013** | 48.43 | 111 | 107772479 |  |  | 24337647 |  |  | 2535958 |  |  |
| **DF014** | 4.27 | 89 | 71745850 |  |  | 70884821 |  |  | 2269851 |  |  |
| **DF016** | 2.32 | 312 | 113390753 | 55507626 |  | 65673569 | 40926903 |  | 2589777 | 2057480 |  |
| **DF019** | 3.02 | 86 | 41809372 |  |  | 50368645 |  |  | 3556444 |  |  |
| **UT001** | 155.11 | 63 | 126638632 |  |  | 91146143 |  |  | 3830431 |  |  |
| **UT002** | 95.46 | 168 | 35400423 |  | 96152939 | 37625489 |  | 38059349 | 5213804 |  | 7106209 |
| **UT003** | 52.44 | 61 | 75484831 |  | 61324575 | 33841014 |  | 37211213 | 2629748 |  | 2936682 |
| **UT005** | 7.01 | 129 | 65868095 |  |  | 36878194 |  |  | 2996522 |  |  |
| **UT006** | 29.38 | 225 | 42277459 |  | 37646534 | 20736119 |  | 26579790 | 2220379 |  | 2128714 |
| **UT007** | 44.24 | 71 | 72284596 | 87147652 |  | 28632693 | 35760057 |  | 1658330 | 2204400 |  |
| **UT008** | 8.85 | 117 | 83971835 |  |  | 63936164 |  |  | 1191323 |  |  |
| **UT013** | 65.24 | 172 | 50899731 |  | 103441134 | 28118836 |  | 30493710 | 2708027 |  | 3160992 |
| **UT015** | 45.49 | 172 | 32857234 |  |  | 50941014 |  |  | 2136414 |  |  |
| **UT016** | 0.08 | N.A. | 77887550 |  | 32439740 | 74955274 |  | 73484164 | 3448562 |  | 1214556 |
| **UT017** | 16.10 | 38 | 140719438 |  | 75587452 | 64730526 |  | 46861463 | 3420625 |  | 8292745 |
| **UT018** | 5.99 | 360 | 41555452 |  |  | 17351611 |  |  | 1617545 |  |  |
| **UT019** | 50.94 | 81 | 100908266 | 116495477 |  | 49537080 | 41790488 |  | 3260336 | 3805953 |  |
| **UT020** | 27.67 | 66 | 73089509 |  | 109190260 | 44300903 |  | 34631761 | 3891076 |  | 5458283 |
| **UT021** | 43.71 | 82 | 83503385 |  | 99004483 | 31875784 |  | 29989158 | 3853632 |  | 2437654 |
| **UT022** | 12.47 | N.A. | 17530452 | 13652130 |  | 20028360 | 17763859 |  | 8620071 | 3177410 |  |
