# Supplementary material for: Circulating metabolites associated with tumor hypoxia and early response to treatment in bevacizumab-refractory glioblastoma after combined bevacizumab and evofosfamide
Source: Front Oncol. 2022 Sep 26;12:900082. doi: 10.3389/fonc.2022.900082 (PMC9549210; doi:10.3389/fonc.2022.900082)
Supplement: Supplementary file 2 [file DataSheet_2.pdf]

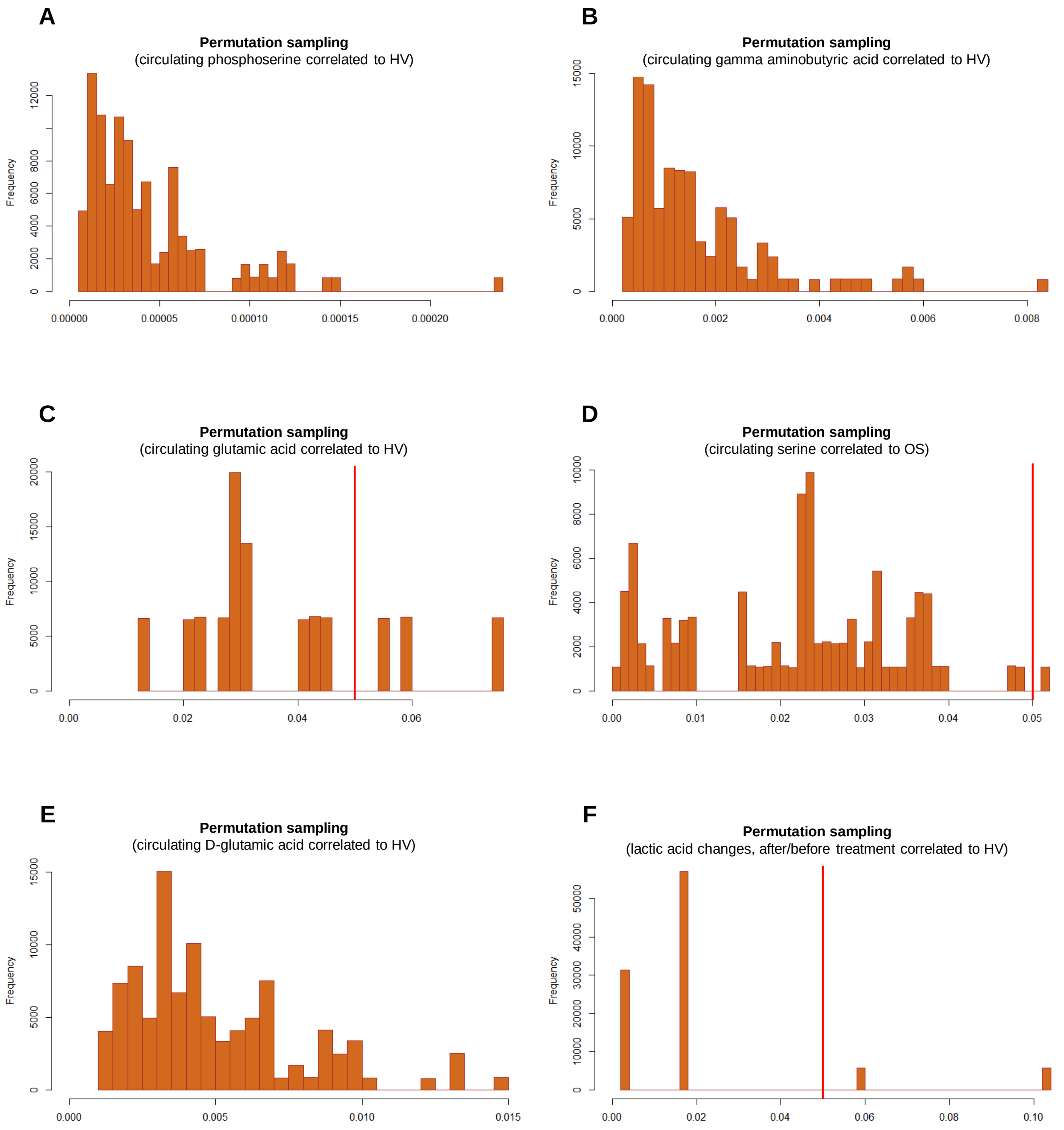

Suppl. Fig. 1. Sample permutation tests support significance of the correlation results. (A-E) Frequency histogram for the 100,000 permutation tests corresponding to (A-E) the levels of metabolites in Fig. 1A-E and (F) the changes of circulating lactate levels following treatment (cycle 2 normalized to before treatment; Fig 2), and their correlation with either HV or OS.
